# Underlying Facets of Cancer Metastasis

**DOI:** 10.3390/cancers14122989

**Published:** 2022-06-17

**Authors:** Abhishek Tyagi

**Affiliations:** Department of Cancer Biology, Wake Forest University School of Medicine, Winston-Salem, NC 27157, USA; atyagi@wakehealth.edu

Even though metastasis, a hallmark of cancer, is responsible for up to 90% of cancer-related mortality, it is still the least understood aspect of cancer pathogenesis [1]. By communicating with the tumor microenvironment (TME), invading cancer cells can overcome stromal challenges, settle, and colonize. It involves intravasation of the primary tumor into the surrounding tissues via the lymph and blood system’s microvasculature, survival, and extravasation into the parenchyma of distant tissues via vascular walls. This invasiveness appears to empower carcinoma cells to form micro-metastatic colonies in the parenchyma, which then proliferate into overt, clinically detectable metastatic lesions (colonization). Additionally, within a cancer cell population, there are heterogeneous clones with intrinsic cellular plasticity that may have a varying degree of metastatic potential. [2]. Furthermore, the tumor microenvironment, inclusive of bulk tumor and tumor stromal cells together with immune cells, fibroblasts, endothelial cells, and pericytes, can also influence cancer progression by producing cytokines that promote or inhibit cancer growth and invasion [3]. As a result, the tumor microenvironment may act as a selective force, just like Darwin’s “natural selection,” to sort out clones that can spread from the primary site to distant sites according to Stephen Paget’s seed and soil hypothesis for cancer spread [4]. Therefore, finding open therapeutic windows for successful interventions necessitates a molecular understanding of the biological mechanisms of the metastatic process, which will allow us to not only understand but also develop novel therapeutic strategies to prevent or treat cancer metastasis in the future.

A central question, yet unaddressed, is the underlying intrinsic characteristics that allow disseminated tumor cells to eventually form overtly metastatic disease. The discovery of metastatic gene signatures, for instance, has undoubtedly added to our understanding of the pathophysiology of metastasis [5]. However, the complexity and inconsistency of a large amount of complex clinical and genomic data available today have raised serious concerns. How should critical genes be chosen to prevent or delay tumor metastatic outgrowth and improve anti-metastatic treatment options? How can metastasis be detected during treatment with high sensitivity and specificity? The first challenge is to determine the functional characterization and mechanism of action of these genes at a molecular level that will inarguably provide true clinical benefit to cancer patients in a precise manner.

Another crucial question is how current treatment affects all the genes and pathways involved in metastasis. Many current patients’ treatment modality is ineffective due to fatal metastasis, despite significant advances and new discoveries in tumor metastasis biology. Importantly, the advancement of new technologies and the development of better tumor models have aided in the discovery of novel gene signatures that predict metastatic spread to specific body sites. Future genetic signature-based clinical studies should be designed with the goal of classifying cancer patients who are likely to develop distant metastases at the earliest possible stage. This will determine which patients will benefit from therapeutic targeting of metastatic-related genes and pathways while avoiding unnecessary treatment, resulting in more cost-effective metastatic treatment that will reduce morbidity and mortality associated with this systemic disease.

Interestingly, earlier research on metastasis has shown cancer stem cells (CSCs) to be a driving force in promoting tumor growth, immune evasion, co-selection of metastatic microenvironments and recurrence in distant organs through their unique biological properties [6]. However, it remains unclear how molecular factors contribute to the CSCs phenotypes, necessitating a mechanistic understanding to improve the metastasis prevention and treatment. Moreover, despite recent evidence suggesting that CSC play a significant role in metastatic colonization [7], effective therapeutic targeting of these cells may be required to better understand and develop more effective and translatable therapies for achieving long-term response during metastasis.

A growing body of evidence also indicates potential role of CSC-derived extracellular vesicles in mediating metastasis, stemness, and remodeling of the tumor immune microenvironment [8]. Understanding the mechanisms of cell communication in the TME mediated by such EVs is therefore crucial for precision therapy targeting CSCs, especially in terms of predicting and preventing future metastatic development to improve cancer patient care. 

Another intriguing aspect of metastasis research that is gaining momentum is the metabolic regulatory crosstalk between CSC and TME in the maintenance of metastasis at distant organs [9,10]. However, the mechanism of metabolic plasticity and TME on CSC have not been fully elucidated, necessitating further research that may reveal novel therapeutic targets.

The articles in this Special Issue are intended to serve as a benchmark for potential mechanistic insights and perspectives on the metastatic process, as well as a reflection on future avenues for developing a framework for potential clinical and translational strategies for cancer metastasis therapy research. We sincerely hope that the articles in this Special Issue will be of interest to the cancers audience, which has dedicated itself to eradicating a threat that is, indeed, awaiting its abolition.
